# Editorial: Insights in Pharmacogenetics and Pharmacogenomics: 2021

**DOI:** 10.3389/fphar.2022.907131

**Published:** 2022-04-14

**Authors:** José A. G. Agúndez, Elena García-Martín

**Affiliations:** Universidad de Extremadura, Institute of Molecular Pathology Biomarkers, University of Extremadura, Cáceres, Spain

**Keywords:** Pharmacogenetics (PGx), Pharmacogenomics (PGx), combinatorial & high-throughput methods, next generating sequencing, clinical practice, NAT1 acetylation, Bac-Mam system

The main aim of this Research Topic is to shed light on recent progress in the Pharmacogenetics and Pharmacogenomics field as well as current and future challenges, aiming to provide a thorough overview of the state of the art in this field.

The research topic comprises 10 articles to which 62 authors have contributed. Throughout the research topic, different aspects related to pharmacological treatment, effectiveness in drug response, and adverse drug events, are covered. Also, relevant aspects in the design of genetic tests are analyzed, regarding the different genetic variants that should be included in pharmacogenomics testing, taking into consideration the ethnicity of the patients. Also, the effects of genetic variants of a relevant enzyme such as the Arylamine N-acetyltransferase 1 (NAT1) in drug metabolism are reviewed, in two aspects: its enzyme activity and the putative influence on other genes that could have consequences beyond drug metabolism such as cancer risk. Finally, methods of expression of genetic variants for *in vitro* functional studies are also assessed.

The review of Tafazoli et al. focuses on the use of next-generation sequencing (NGS) in guiding drug treatment in clinical practice. It discusses both, the limitations of NGS platforms and putative solutions for solving these limitations. The reduced cost, one of the most important aspects, together with the capacity of simultaneously analyzing a large number of pharmacogenes, as well as the deep analysis it provides, makes this technology promising for routine clinical use.

The article by Sayer et al. analyzes the detection rate of currently available Combinatory Pharmacogenomics tests (tests covering multiple genes) (Wilke et al., 2005; Winner and Dechairo, 2015; Brown et al., 2017), which is widely used in recent years because of their economic utility, in this case, based on the cytochrome P450 gene variants they target. One interesting point in this study addresses the differences in the frequencies of genetic variants between different ethnic groups can make available tests less effective: The authors conclude that the detection rate of CPGx tests covering *CYP2C19*, *CYP2C9*, *CYP2D6*, and *CYP2B6*, show significant variation across ethnic groups, Sub-Saharian Africans and East Asians have a high rate of incorrect detection.

Two articles in this research topic addressed new clinical applications of pharmacogenetics. The work by Hongkaew et al. focuses on the treatment of autism spectrum disorder with risperidone. The authors created an algorithm to calculate the DRD2 genetic risk based on inferred protein expression. The article by Sales et al. is a systematic review assessing the utility of pharmacogenomics in the treatment of sickle cell anemia (SCA) with hydroxyurea. They analyzed more than 700 SNVs and identified 50 SNVs associated with fetal hemoglobin changes in patients with SCA treated with hydroxyurea.

Two articles on this research topic put the focus on the NAT1 (arylamine N-acetyltransferase1) enzyme. The work by Carlisle et al. studied the effect of NAT1 expression levels on the expression of other genes. They found that nearly 3,900 genes were significantly associated with NAT1 activity in breast cancer cell lines modified to have increased, decreased, and null levels of NAT1. Thus, their work shows that NAT1 activity causes expression changes in many genes, thus raising the possibility that the role of NAT1 in cancer (Hein, 2000; Agundez, 2008; Carlisle et al., 2018) could be ultimately mediated by genes other than *NAT1*. The study by Doll and Hein analyzes the effects of SNVs in the *NAT1* coding exon on Michaelis-Menten kinetic constants for the carcinogen 4-aminobiphenyl and its N-hydroxylated metabolite, that are NAT1 substrates (Hein et al., 1993). They confirmed that some SNVs significantly reduced acetyltransferase activity. Also, they identified an SNV (rs4986782) that significantly reduced the apparent Km for these carcinogens.

The article by Muderrisoglu et al. analyzes the effects of SNVs in genes coding human nicotinic acetylcholine receptor subunits on nicotine addiction and the efficacy of smoking cessation therapy with varenicline, nicotine replacement therapy or bupropion. The study concludes that the response to smoking cessation treatments is independent of the nicotinic acetylcholine receptor α subunits genotype analyzed.

Giles et al. reviewed the applicability of several omic techniques (genomics, metagenomics, transcriptomics, proteomics, and metabolomics), in heparin-induced thrombocytopenia (HIT), an adverse drug event that has a high mortality (Jolicoeur et al., 2009). The study covers the relevance and current knowledge on omics in HIT, and it stresses the importance of multi-omics approaches to gain ground on the pathogenesis of this adverse drug event.

The aim of the study by Gloor et al. is to identify genetic risk factors for postoperative nausea and vomiting (PONV), aiming to improve the identification of at-risk patients. For this, they selected SNVs located around 13 different genes, including COMT, CHRM3, five 5-hydroxytryptamine receptors (HTR) subunits, OPRM1, DRD2, TACR1, FAAH1, ABCB1, as well as the inferred phenotypes for the cytochrome P450 enzymes CYP2D6, CYP3A, CYP2C9, CYP2C19, CYP1A2, and CYP2B6. Interestingly, they identified association or PONV recurrence with the CYP1A2 activity score and with *TACR1* and *HTR3* genotypes, and they developed a risk model based on these factors.

Finally, Miyauchi et al. analyzed the potential of the baculovirus-mammalian cell (Bac-Mam) expression system to analyze the effect of genetic variability in drug metabolism. This technique allows the transference of genes coding for drug-metabolizing enzymes into mammalian cells to obtain correct posttranslational modifications. The authors demonstrated that *CYP3A4*, *UGT1A1*, and *UGT2B7* can be efficiently transfected using this procedure. The Bac-Mam expression system, therefore, holds great promise for the functional analysis of the plethora of SNVs and allelic variants present in pharmacogenes, whose list is in continuous expansion as new genetic variations are described.

In sum, this Research Topic covered interesting findings and procedures that will help to gain ground in the development of Pharmacogenetics and Pharmacogenomics and their implementation in clinical practice.
